# Evaluating the Effectiveness of Commercial Oral Supplements for Hair Growth: A Systematic Review and Meta‐Analysis

**DOI:** 10.1111/jocd.70817

**Published:** 2026-04-09

**Authors:** Razan Alanazi, Shadan Alshammari, Kadi Aldossari, Raneem Alwatban, Deema Aljeribah, Shoog Alanazi, Abdulaziz Alsalhi, Aminah Alhumam, Abdulmajeed Alajlan

**Affiliations:** ^1^ College of Medicine University of Hail Hail Saudi Arabia; ^2^ College of Medicine King Saud University Riyadh Saudi Arabia; ^3^ Dermatology Consultant, Department of Dermatology University of British Columbia Vancouver BC Canada; ^4^ College of Medicine King Faisal University Al‐Ahsa Saudi Arabia; ^5^ Professor, Department of Dermatology, College of Medicine King Saud University Riyadh Saudi Arabia

**Keywords:** alopecia, androgenetic alopecia, hair growth, nutraceuticals, oral supplements, telogen effluvium

## Abstract

**Background:**

Hair loss is a common condition affecting both men and women, with significant psychosocial impact. While oral supplements are marketed to improve hair growth, their clinical efficacy remains debated. This systematic review aimed to evaluate the effectiveness and safety of commercial oral nutraceuticals for hair growth.

**Methods:**

For this systematic review, a comprehensive search was undertaken in PubMed, Embase, Cochrane CENTRAL, and Web of Science. Eligible studies included randomized controlled trials (RCTs), open‐label trials, and proof‐of‐concept studies evaluating oral supplements in adults (≥ 18 years) with alopecia or hair thinning. Primary outcomes included objective measures including hair counts, density, growth rate, tensile strength, and pull test. Secondary outcomes were investigator/patient assessments and adverse events.

**Results:**

A total of 14 studies, including 967 participants, were included. Supplements assessed included Ceramosides, Nutrafol, Nourkrin, Lambdapil, Viviscal, Forti5, GFM oral supplement, and Cynatine HNS. Most trials were RCTs with durations of 12 weeks to 12 months. Meta‐analysis demonstrated significant improvements with oral supplements versus placebo: reduced telogen hair density (mean difference [MD] −5.91, 95% confidence interval [CI] −6.74 to −5.08, *p* < 0.00001), increased anagen hair density (MD 10.48, 95% CI: 8.81 to 12.14, *p* < 0.00001), and reduced telogen proportion (MD 3.08, 95% CI: −3.35 to −2.81, *p* < 0.00001). Similarly, significant differences were observed regarding anagen proportion, elongation, and hairs extracted on a pull test (*p* < 0.00001 for all). Total hair count showed no significant difference (*p* = 0.06). Patient‐reported outcomes consistently favored supplements, reporting improved thickness, reduced shedding, and better satisfaction. Adverse events were minimal, and safety profiles were favorable.

**Conclusions:**

In conclusion, supplements show promising preliminary results, but more rigorous, independent research is needed to confirm these findings.

## 1 Introduction

Hair is a prominent aspect of personal appearance and identity, and loss of hair can greatly affect self‐image and well‐being [1]. Androgenetic alopecia (AGA), or pattern hair loss, is the most common form of non‐scarring hair loss, affecting the majority of men and a substantial proportion of women over the lifespan [2]. This type of hair loss affects approximately 80% of men and about 50% of women by late adulthood [3]. Other non‐scarring forms of hair loss include telogen effluvium (TE) and alopecia areata [4]. Multiple factors contribute to these conditions including genetics, hormonal milieu, nutritional status, illness, and medications [5]. For example, severe metabolic stress or nutritional deficiency can precipitate TE, while dihydrotestosterone (DHT)‐mediated miniaturization underlies AGA [2]. Given the substantial psychosocial burden of hair loss, many affected individuals turn to over‐the‐counter supplements in hopes of improving hair growth. The global market for hair growth supplements has expanded rapidly; however, market growth reflects consumer demand rather than proven clinical efficacy. One recent analysis projected the market value at USD 2.86 billion by 2031, which reflects strong consumer demand [6]. Trends in Internet search data mirror this growth. For instance, searches for common supplements such as topical minoxidil, biotin, and collagen have risen sharply over the past decade [7]. However, heightened public interest does not necessarily correlate with high‐quality evidence supporting effectiveness [8].

This booming market is driven by the perception of natural supplements as attractive alternatives to prescription drugs, especially given the limited FDA‐approved options for hair loss. Topical minoxidil is the only treatment FDA‐approved for both male and female pattern hair loss, and oral finasteride (a 5α‐reductase inhibitor) is approved for men [9]. However, minoxidil's efficacy is modest and it can cause scalp irritation or unwanted facial hair while finasteride often incurs sexual side effects that deter some patients [9]. Due to these limitations, many individuals seek alternative or adjunctive remedies. Nutraceuticals, broadly defined as oral supplements containing vitamins, minerals, amino acids, fatty acids, botanical extracts, or other bioactive compounds, have emerged as popular interventions for hair growth [10]. However, claims that these products nourish hair follicle systemically are largely theoretical and not uniformly supported by robust clinical data [11]. Examples of commonly promoted ingredients include biotin (vitamin B7), zinc, saw palmetto, omega‐3/6 fatty acids, tocotrienols, and various herbal extracts [9, 12]. Several clinical trials have suggested that certain vitamins, omega fatty acids, and antioxidants can modestly improve hair count or density [12, 13, 14]. However, the overall quality of evidence reported in these studies is heterogeneous, with small sample sizes and inconsistent outcome measures.

Several other pharmacological interventions have been used in clinical practice for AGA including dutasteride [15], low‐dose oral minoxidil [16], and spironolactone [17] but these are largely off‐label. Procedural options such as hair transplantation also represent established, evidence‐based treatments for AGA patients [18]. Similarly, multi‐ingredient supplements like marine collagen complexes, wheat protein extracts, herbal blends, and keratin‐based formulas have reported favorable outcomes in studies [9, 19]. Despite the widespread use of these nutraceuticals, their regulatory oversight and study quality vary, and the overall evidence base remains mixed and somewhat unclear. Given the proliferation of hair growth supplements and the variable trial results, a systematic synthesis of the evidence is needed. Therefore, this systematic review was conducted to investigate the efficacy of commercial oral supplements for hair growth.

## 2 Methods

This systematic review was conducted in accordance with the guidelines outlined in the Cochrane Handbook for Systematic Reviews of Interventions. A predetermined protocol was prospectively registered in the International Prospective Register of Systematic Reviews (PROSPERO) under registration number CRD420251059749

### 2.1 Eligibility Criteria and PICOS Framework

All randomized controlled trials published through July 2025 were eligible for inclusion. The review further adhered to the Preferred Reporting Items for Systematic Reviews and Meta‐Analyses (PRISMA) guidelines [20]. As each individual study had already obtained ethical approval, no additional institutional review board permission was required for this review. The PICOS framework for this review was defined as follows: the population comprised adults aged 18 years or older with self‐perceived or clinically diagnosed hair thinning or loss, including androgenetic alopecia, telogen effluvium, or chronic shedding; the intervention was oral nutraceutical or supplement formulations targeting hair growth or quality; the comparator included placebo, no treatment, or active drug co‐treatment; the outcomes included quantitative hair parameters such as terminal, vellus, and total hair counts, hair density, anagen‐to‐telogen ratios, hair growth rate, diameter, tensile strength, pull‐test results, investigator or photographic assessments, patient‐reported scales including visual analogue scales and quality‐of‐life questionnaires, and adverse events; and the study design included randomized controlled trials (parallel‐group, double‐blind, assessor‐blinded), open‐label prospective studies, and proof‐of‐concept trials.

### 2.2 Search Strategy and Data Sources

An experienced medical librarian collaborated to develop comprehensive search strategies for PubMed, Embase, Cochrane CENTRAL, and Web of Science from database inception through July 2025. Searches combined controlled vocabulary (e.g., MeSH) and keywords related to hair loss (“alopecia,” “hair thinning,” “effluvium”) with terms for nutraceutical interventions (“oral supplement,” “nutraceutical,” “marine extract,” “wheat lipid complex”). No language or date restrictions were applied at the search stage. Reference lists of included studies and pertinent reviews were hand‐searched to capture any additional eligible trials.

### 2.3 Study Selection

The search results from databases were transferred to the reference manager (EndNote 20, Thomson Reuters). The results were combined and uploaded to Rayyan, a web‐based software [21]. Before screening, duplicates were removed. During the screening process, four independent reviewers were involved. The reviewers were divided into two groups, with group A involving two reviewers (SA and KA) and group B including two reviewers (RW and DA). During the screening process, the blind was turned on in Rayyan to minimize bias during the selection process. Firstly, the records were screened based on title and abstract. In the second step, the blind was removed and the selection was compared. A final decision was made after discussion. In case of any disagreement, a fifth reviewer (RA) was involved. Next, full‐length screening was performed. Finally, the data was extracted from the included studies in an Excel file regarding patient demographics and patient outcomes.

### 2.4 Risk‐of‐Bias Assessment

Risk‐of‐bias assessment was conducted using the Cochrane Risk of Bias 2.0 (RoB 2) tool for randomized controlled trials, evaluating domains including the randomization process, deviations from intended interventions, missing outcome data, outcome measurement, and selective reporting. For non‐randomized and open‐label studies, the Methodological Index for Non‐Randomized Studies (MINORS) was applied, addressing aspects such as study aim, patient inclusion, prospective data collection, appropriate endpoints, unbiased outcome assessment, adequate follow‐up, and sample size calculation. Two reviewers independently performed the assessments, and any disagreements were resolved through consensus or by consulting a third reviewer.

### 2.5 Outcome Measures

Primary outcomes included objective hair parameters: terminal, vellus, and total hair counts; hair density (hairs/cm^2^); anagen and telogen proportions; hair growth rate; hair diameter and tensile strength; and pull‐test results. Secondary outcomes encompassed investigator or photographic global assessments and patient‐reported measures (visual analogue scales for hair density or volume; quality‐of‐life questionnaires). Safety outcomes comprised adverse events and tolerability assessments.

### 2.6 Data Analysis

A random‐effects model was applied using the DerSimonian‐Laird technique [22]. This method incorporates variability between studies and generates a pooled estimate along with a 95% confidence interval (CI). Mean difference was selected as the measure of effect. Differences among trials were examined with the *I* ^2^ index, which expresses the proportion of variance due to heterogeneity instead of chance. An *I* ^2^ value above 50% was considered to indicate meaningful inconsistency. Results of the synthesis are presented as forest plots, showing the combined estimates and their 95% CIs. Statistical importance was assessed through *p*‐values, with a cutoff of 0.05.

## 3 Results

### 3.1 Included Studies

The literature search provided 700 studies from the PubMed (*n* = 340), Cochrane (*n* = 104), and CINAHL (*n* = 256). During screening, 448 records were removed based on title and abstract. 17 articles were included in the full‐length screening. Following a thorough assessment of full‐length articles, 14 articles were included in this systematic review. Figure S1 shows the PRISMA flow diagram of this systematic review.

### 3.2 Study Characteristics

A total of 967 participants were included in 14 studies. Two studies were prospective studies whereas the remaining were RCTs. The ages of included participants ranged from 18 years to 71 years. Interventions were oral nutraceuticals and supplements such as Ceramosides, Nutrafol, Nourkrin, a GFM supplement, Lambdapil‐ISDIN, Viviscal, Forti5, Cynatine HNS, and fish extract; comparators were usually placebo or, in one trial, an active fish extract [23]. Dosing schedules varied by product and study durations spanned from 12 weeks up to 12 months (Table S1).

Most trials reported statistically significant improvements in hair growth parameters compared with placebo, including increased anagen hair percentage, higher terminal hair counts, improved hair density and volume, and reduced shedding. For example, Dudonné et al. found a 23.5% reduction in telogen hairs and a 12.5% increase in growth with Ceramosides [24] while Bhatia et al. reported improved growth and quality scores with Nutrafol Men's Capsules and significant stress reduction [25]. PROs generally indicated high satisfaction rates, perceived improvements in growth, thickness, and coverage, and better QoL scores. Only Piquero‐Casals et al. [26] found no significant difference in QoL despite objective gains. Safety profiles were favorable across interventions, with no or minimal adverse events reported (Table S2).

### 3.3 Bias Assessment

**FIGURE 1:**
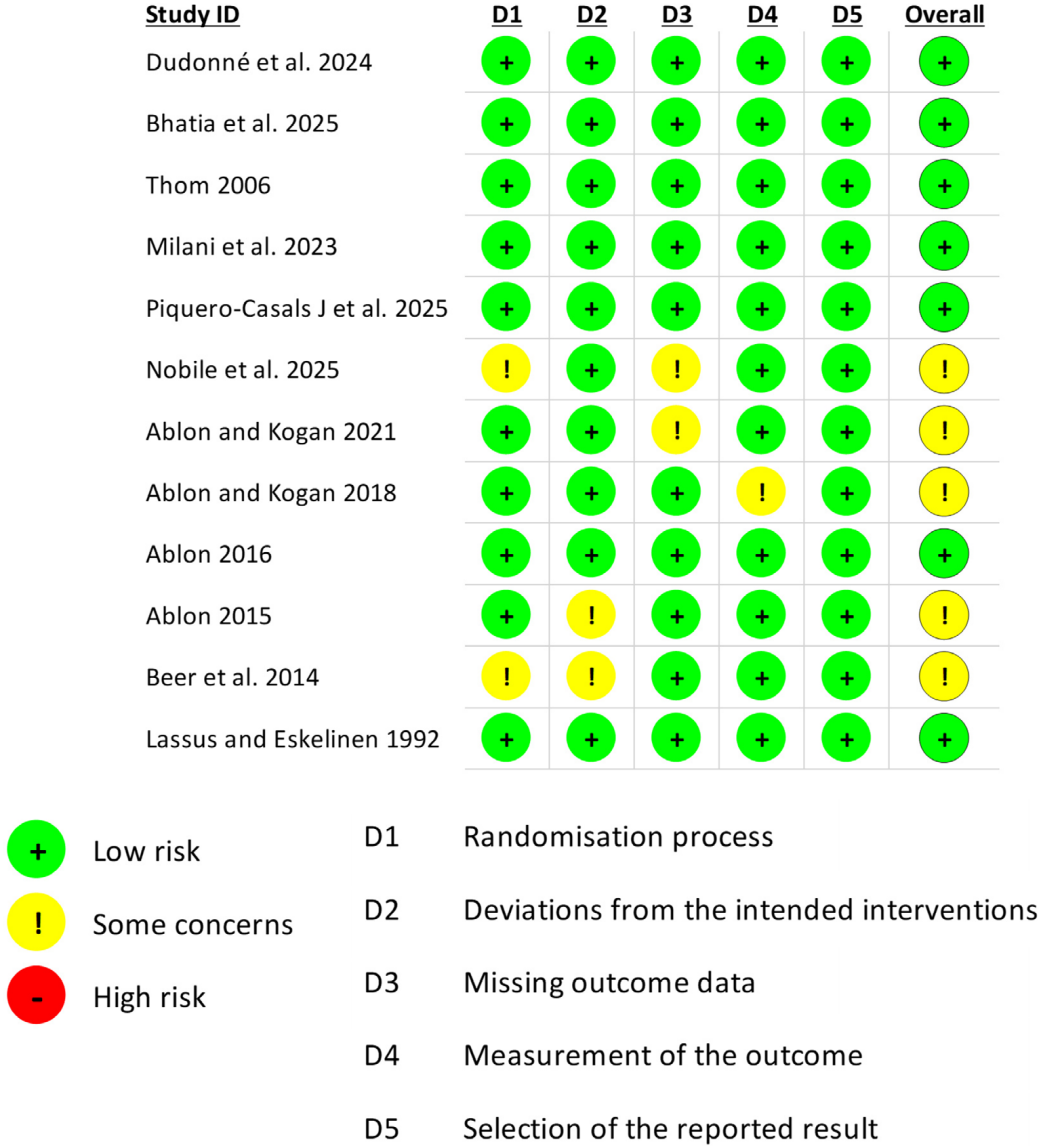
Risk of bias assessed with ROB‐2.

Across the 12 studies assessed with RoB‐2, most trials showed low risk of bias overall: 7 studies were judged low risk, 5 studies had “some concerns,” and none were rated high risk. At the domain level, concerns were most often seen in the randomization process (2 studies), deviations from intended interventions (2 studies), and missing outcome data (2 studies); one study had concerns about outcome measurement and none showed concerns for selective reporting (Figure 1).

Risk of bias for non‐randomized studies was measured with the MINORS tool. Sivamani et al. [27] had a moderate level of risk of bias (11/16), whereas Nichols et al. [28] had a low risk of bias (14/16). Sivamani et al. [27] had a high risk regarding the inclusion of consecutive patients. Both studies had a high risk of bias pertaining to the prospective calculation of study size (Table 1).

**TABLE 1:** Risk of bias assessed with MINORS tool.

No.	Item	Sivamani et al. [27]	Nichols et al. [28]
1	A clearly stated aim	2	2
2	Inclusion of consecutive patients	0	2
3	Prospective data collection	2	2
4	Endpoints appropriate to the aim of the study	2	2
5	Unbiased assessment of the study endpoint	2	2
6	Follow‐up period appropriate to the aim of the study	2	2
7	Loss to follow‐up less than 5%	1	2
8	Prospective calculation of the study size	0	0

### 3.4 Telogen Hair Density (Hair/cm^2^)

**FIGURE 2:**
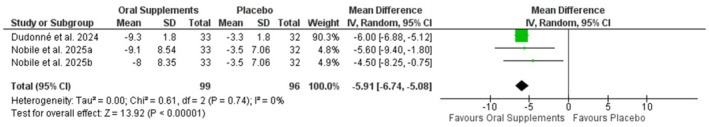
Forest plot of telogen hair density (hair/cm^2^).

Telogen hair density (hair/cm^2^) was reported in three studies [24, 29]. Compared to placebo, telogen hair density was significantly lower in the oral supplements group (MD −5.91, 95% CI: −6.74 to −5.08, *p* < 0.00001). Statistical heterogeneity was low (*I* ^2^ = 0%) (Figure 2).

### 3.5 Anagen Hair Density (Hair/cm^2^)

**FIGURE 3:**
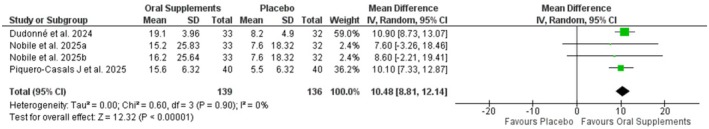
Forest plot of anagen hair density (hair/cm^2^).

Anagen hair density (hair/cm^2^) was reported in four studies [24, 26, 29]. Compared to placebo, anagen hair density was significantly higher in the oral supplements group (MD 10.48, 95% CI: 8.81 to 12.14, *p* < 0.00001). Statistical heterogeneity was low (*I* ^2^ = 0%) (Figure 3).

### 3.6 Telogen Hair Proportion (%)

**FIGURE 4:**
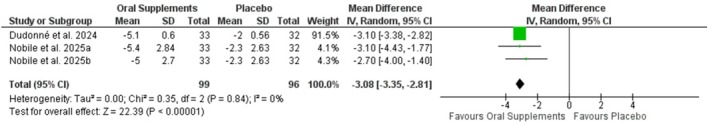
Forest plot of telogen hair proportion (%).

Only three studies reported telogen hair proportion (%) [24, 29]. Oral supplement group had significantly lower telogen hair proportion compared to placebo (MD −3.08, 95% CI: −3.35 to −2.81, *p* < 0.00001). Statistical heterogeneity was low (*I* ^2^ = 0%) (Figure 4).

### 3.7 Anagen Hair Proportion (%)

**FIGURE 5:**
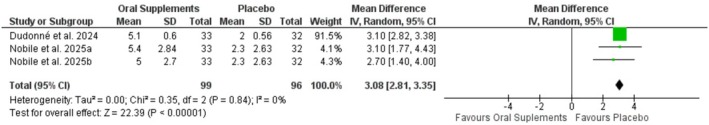
Forest plot Anagen hair proportion (%).

Anagen hair proportion (%) was reported in three studies [24, 29]. Compared to placebo, anagen hair proportion (%) was significantly higher in oral supplements (MD 3.08, 95% CI: −2.81 to 3.35, *p* < 0.00001). Statistical heterogeneity was low (*I* ^2^ = 0%) (Figure 5).

### 3.8 Hair Pull Test

**FIGURE 6:**
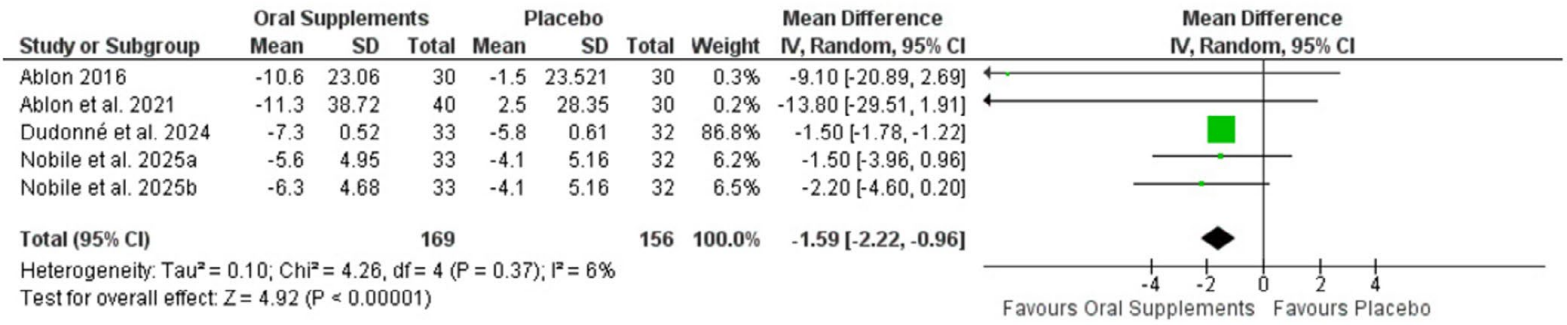
Forest plot of hair pull test.

Five studies reported number of hairs pulled on hair pull test [24, 29, 30, 31]. Participants in oral supplement groups had significantly lower hairs on pull test compared to placebo (MD −1.59, 95% CI: −2.22 to −0.96, *p* < 0.00001). Statistical heterogeneity was low (*I* ^2^ = 6%) (Figure 6).

### 3.9 Elongation (% Of Initial Length)

**FIGURE 7:**
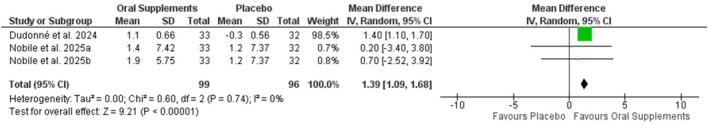
Forest plot of Elongation (% of initial length).

Elongation (% of initial length) was reported in three studies [24, 29]. Compared to placebo, elongation (% of initial length) was significantly higher in oral supplements (MD 1.39, 95% CI: 1.09 to 1.68, *p* < 0.00001). Statistical heterogeneity was low (*I* ^2^ = 0%) (Figure 7).

### 3.10 Total Hair Count

**FIGURE 8:**
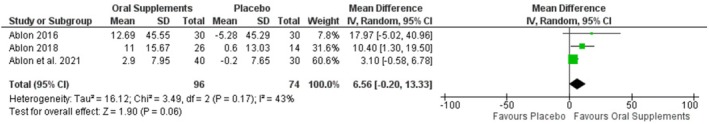
Forest plot of total hair count.

Total hair count was reported in three studies [24, 29]. No significant difference was observed between oral supplements and placebo regarding total hair count (MD 6.56, 95% CI: −0.2 to 13.33, *p* = 0.06). Statistical heterogeneity was moderate (*I* ^2^ = 43%) (Figure 8).

## 4 Discussion

Despite the substantial clinical heterogeneity across the diverse supplement formulations, the pooled analysis based on evidence from 14 studies including 967 participants revealed improvements in hair growth parameters for supplement users compared to placebo. Outcome measures such as telogen and anagen hair density, telogen and anagen hair proportion, number of hairs on hair pull test, and elongation percentage from initial length showed improvement in oral supplement treatment groups compared to placebo in included studies in meta‐analysis. Despite the fact that only limited studies reported data that could be included in meta‐analysis, all studies reported improvements in their measured outcomes in patients treated with various oral supplements compared to those who did not receive intervention treatment. Although several outcomes reached statistical significance (*p* < 0.05), most were based on only 2–4 trials. Such small numbers increase susceptibility to small‐study effects and imprecision. Several included trials were funded or supported by supplement manufacturers. Industry sponsorship introduces potential risk of bias, including selective reporting and emphasis on favorable endpoints.

While statistical heterogeneity (*I* ^2^) was low for most outcomes, significant clinical heterogeneity was present due to variations in supplement formulations, dosages, treatment durations, and patient populations (e.g., different types of alopecia, age, gender). This clinical heterogeneity suggests that our pooled estimates should be interpreted with caution, as they represent an average across diverse interventions and conditions. We were unable to perform planned subgroup analyses (e.g., by supplement type, gender, baseline condition) due to insufficient primary data. Future individual participant data meta‐analyses are needed to explore these effect modifiers.

Most studies reported double‐digit improvements in outcomes. For example, Dudonné et al. reported that women taking a wheat polar lipid complex (Ceramosides) had a 23.5% reduction in telogen hair and an 11.9% increase in anagen hair after 12 weeks, versus far smaller changes with placebo [24]. Similarly, Nobile et al. observed that acute telogen effluvium patients given Ceramosides oil or powder had up to 26%–27% decreases in telogen hair and 10%–11% increases in anagen hair, significantly more than the 5.4% increase seen with placebo [29]. Furthermore, some phototrichogram analyses and global photography assessments also corroborated findings. In the RCT of Bhatia et al. 79% of men on Nutrafol Men's supplement were rated improved in hair growth versus 51% on placebo (*p* < 0.01) [25]. Similarly, a marine proteoglycan supplement showed a significant positive effect on hair growth measures in a 6‐month RCT [32].

Patient‐reported outcomes and satisfaction also aligned with objective findings. High proportions of participants on active treatment reported perceptible benefits. In Bhatia et al. 83% of men taking Nutrafol Men's reported satisfaction with hair growth versus 66% on placebo [25]. The Men's Hair Growth Questionnaire (MHGQ) in their trial showed significantly higher positive responses on measures of slowed hair loss and scalp appearance with active supplement. Similarly, Dudonné et al. found that 64% of women on Ceramosides felt their hair density and volume had improved, compared to 53%–59% on placebo [24]. Ablon and Kogan noted that 73%–81% of women on Nutrafol Women's Capsules reported perceived growth, volume, or thickness improvements in a self‐assessment questionnaire [33]. Quality‐of‐life (QoL) and visual analogue scales, when measured, tended to improve with supplements, though some studies did not find statistically significant QoL differences despite objective gains. No study reported worsening of QoL or notable distress from taking the supplements. Furthermore, safety and tolerability were uniformly excellent. Across trials, oral supplements caused few if any adverse events. No moderate or severe adverse events were attributed to the interventions, and dropout rates were low. For instance, Bhatia et al. explicitly reported no trends in sexual dysfunction with Nutrafol Men's and no significant differences in adverse effects between supplement and placebo groups [25]. This favorable safety profile is consistent with other reviews noting that nutraceuticals for hair loss tend to be well tolerated [19]. The favorable short‐term safety profile reported across trials is encouraging. However, the longest follow‐up in our included studies was 12 months, and adverse events were often collected passively. The long‐term safety of continuous, high‐dose intake of many botanical ingredients remains uncertain. We emphasize the need for future studies with active surveillance over extended periods (≥ 2 years) to rule out rare or delayed adverse events, particularly those related to hormonal effects (e.g., from saw palmetto) or interactions with medications.

The findings of the present systematic review are consistent with prior literature on efficacy of oral hair supplements. For instance, a review by Ring et al. concluded that oral nutraceuticals produce modest hair growth in androgenetic alopecia patients [9]. Similarly, in a systematic review of 30 studies on nutritional interventions for hair loss in individuals without baseline deficiencies found that most evidence showed improvements in hair growth outcomes. The strongest evidence supported Viviscal, Nourkrin, Nutrafol, Lamdapil, Pantogar, capsaicin with isoflavone, omega‐3/6 with antioxidants, apple nutraceutical, paeony with glycyrrhizin, zinc, tocotrienol, and pumpkin seed oil. Lower‐quality evidence suggested possible benefits of kimchi and cheonggukjang, vitamin D3, and Forti5 [19]. The observed clinical benefits are supported by emerging mechanistic data. For instance, marine‐derived complexes have been shown in vitro to upregulate genes like β‐catenin and VEGF, which are pivotal for anagen phase induction and follicular angiogenesis [34]. Similarly, saw palmetto may exert anti‐androgenic effects by inhibiting 5α‐reductase, albeit more weakly than finasteride. Antioxidants combat oxidative stress in the follicle which is implicated in hair follicle miniaturization. Future research should integrate clinical outcomes with biomarker assessments (e.g., scalp DHT levels, inflammatory cytokines) to further elucidate these mechanisms in humans. The efficacy outcomes reported in the present systematic review and previous systematic review can be explained by the nutritional and biochemical roles of supplement ingredients. Many hair supplements contain biotin, vitamins (C, D, E), minerals (zinc, iron), amino acids (cysteine, keratin), and herbal extracts that are known to support the hair growth cycle. For example, iron and vitamin D are cofactors in cell proliferation, and deficiencies are linked to telogen effluvium [13].

In our included trials, even participants without overt nutritional deficiencies often improved, possibly because subclinical insufficiencies are common or high doses exert pharmacological effects. Pumpkin seed oil, rich in β‐sitosterol, may inhibit 5α reductase, mimicking the action of finasteride but with fewer side effects. An RCT by Cho et al. evaluated the efficacy of 400 mg/day pumpkin seed oil (PSO) in 76 men with mild to moderate androgenetic alopecia over 24 weeks. The PSO group showed significantly greater improvements in self‐rated improvement (*p* = 0.013) and satisfaction (*p* = 0.003) compared to placebo. Hair counts increased by 40% in the PSO group versus 10% in placebo (*p* < 0.001), with no difference in reported adverse effects between groups [35]. Marine‐derived supplements such as Viviscal and Nourkrin supply glycosaminoglycans and proteins that could support follicle matrix formation.

A study by Augustyniak and Mahon evaluated the hair growth‐promoting effects of marine‐derived ingredients contained in the supplement Viviscal and its components (AminoMarC, shark and oyster extract) using dermal papilla cell models. The compounds showed significantly increased dermal papilla cell proliferation. They also enhanced production of alkaline phosphatase and glycosaminoglycans, alongside upregulation of genes linked to the hair cycle [36]. These findings suggest that marine‐derived ingredients may promote hair growth by stimulating anagen activation. Research has identified follicular proteoglycans such as versican and decorin as key regulators of hair cycle transitions, with reduced expression linked to anagen shortening and follicular miniaturization [37]. Clinical studies of proteoglycan replacement therapy (PRT) with an oral marine‐derived extract (Nourkrin with Marilex) demonstrated significant benefits, including reduced hair fall, increased hair growth, and improved quality of life in both male and female patients [37]. These mechanistic considerations lend biological plausibility to the observed effects in this systematic review.

## 5 Strengths and Limitations

This review has several strengths. First, a systematic search was undertaken in various databases and the protocol of the systematic review was registered on PROSPERO. Second, the systematic review strictly adheres to PRISMA guidelines. To ensure that maximum evidence is included in the systematic review, prospective studies were also included apart from RCTs. Furthermore, for outcomes that were reported in multiple studies, meta‐analysis was also conducted. However, there are certain limitations of this systematic review that should be considered while interpreting findings. The main limitation of this review is the small evidence base as relatively few trials contributed data to each outcome. Another limitation of this systematic review is the concerns related to publication bias. Although we acknowledge the potential for publication bias, a formal statistical assessment using funnel plots or Egger's test was not feasible due to the limited number of studies (< 10) for each outcome meta‐analyzed. This limitation increases the risk that small, negative trials remain unpublished, potentially inflating the overall effect estimates observed in our review. Future updates with a larger number of studies should include such assessments. All included studies report positive or null findings, and negative trials (if any) may be unpublished. We did not formally assess publication bias via funnel plots due to the small number of studies per outcome. The clinical heterogeneity including different products, dosages, patient populations means that our pooled effect estimates should be viewed cautiously. In reality, each supplement likely has a distinct efficacy profile. Although statistical heterogeneity was low for most outcomes, this may reflect insufficient data rather than true uniformity. Furthermore, we did not have access to individual patient data, which limits subgroup analysis. For several hair parameters such as tensile strength, hair diameter, and some phototrichogram measures, there are few or no independent replications. This means the findings should be regarded as preliminary and exploratory rather than confirmatory. Another limitation was that the follow‐up duration was short in most studies.

## 6 Implications for Practice and Research

Despite certain limitations of this systematic review, current findings have implications for practice. For clinicians and patients, this review suggests that oral hair supplements can be considered as adjunctive therapy for hair thinning, with the expectation of modest improvement. Given the favorable safety profile, discussing them as part of shared decision‐making seems reasonable. However, patients should be counseled that supplements are not a cure. Hair gains are incremental and typically require continuous use for several months before assessing benefit. In practice, combining nutraceuticals with proven treatments such as minoxidil and finasteride might yield synergistic effects, though data on such combinations are lacking. Importantly, practitioners should inquire about existing vitamin/mineral levels. If a patient has iron deficiency or vitamin D insufficiency, correcting those deficiencies is known to help hair growth [13] and may even be more effective than over‐the‐counter supplements alone. However, it is also important to note that quality control of supplements is variable, as they are not FDA‐regulated like drugs. Consumers should be advised to use reputable brands and to be wary of unsubstantiated marketing claims. Despite encouraging evidence, many questions remain. Longer and larger RCTs are needed to confirm durability of benefit and optimal dosing. Most trials to date have been industry‐supported and single‐center. Thus, truly independent, multicenter studies would strengthen confidence. Further elucidation of mechanisms on how specific botanicals influence the hair follicle will help refine formulations. Importantly, future trials should use standardized outcome measures such as validated hair count methods and uniform QoL scales to facilitate comparisons.

## 7 Conclusion

In summary, this systematic review found that commercial oral nutraceuticals for hair growth generally produce modest but significant improvements in hair density, thickness, and shedding compared to placebo. These improvements were observed across multiple formulations and patient populations. However, evidence provided in included studies is heterogeneous. Formulations, ingredient combinations, doses, follow‐up durations, and participant characteristics varied widely. Many included trials were small, of short duration, and several reported industry sponsorship. Although adverse events were generally few in the included trials, follow‐up periods were short and adverse‐event ascertainment was often passive or inconsistently reported. Currently, there is a need for high quality, independently funded randomized trials with longer follow‐up, standardized outcome measures. The studies should focus on subgroup analysis to define which agents are effective, for whom, and with what safety profile.

## Author Contributions

**Razan Alanazi:** conceptualization, methodology, project administration, screening conflict resolution, writing – original draft, supervision. **Shadan Alshammari:** investigation, screening (Title/Abstract and Full‐Text), data curation, formal analysis, writing – review and editing. **Kadi Aldossari:** investigation, screening (Title/Abstract and Full‐Text), data curation, validation, writing – review and editing. **Raneem Alwatban:** investigation, screening (Title/Abstract and Full‐Text), data curation, validation, writing – review and editing. **Deema Aljeribah:** investigation, screening (Title/Abstract and Full‐Text), data curation, validation, writing – review and editing. **Shoog Alanazi:** writing – review and editing. **Abdulaziz Alsalhi:** supervision, validation, writing – review and editing. **Aminah Alhumam:** writing – review and editing. **Abdulmajeed Alajlan:** supervision, resources, validation, writing – review and editing. Screening Groups: Group A: Shadan Alshmmari and Kadi Aldossari. Group B: Raneem Alwatban and Deema Aljeribah. Fifth Reviewer (Conflict Resolution): Razan Alanazi. All authors have read and approved the final manuscript.

## Conflicts of Interest

The authors declare no conflicts of interest.

## Supporting information


**Figure S1:** PRISMA flow diagram showing the study selection process.

**Table S1:** Detailed characteristics of the 14 included studies, including study design, population, intervention, comparators, and study duration.
**Table S2:** Summary of efficacy findings, patient‐reported outcomes (PROs), and safety profiles of the evaluated oral nutraceuticals.

## Data Availability

Data sharing not applicable to this article as no datasets were generated or analysed during the current study.
