# Supplementary figures and images for: Evaluating the Effectiveness of Commercial Oral Supplements for Hair Growth: A Systematic Review and Meta‐Analysis
Source: J Cosmet Dermatol. 2026 Apr 9;25(4):e70817. doi: 10.1111/jocd.70817 (PMC13063204; doi:10.1111/jocd.70817)

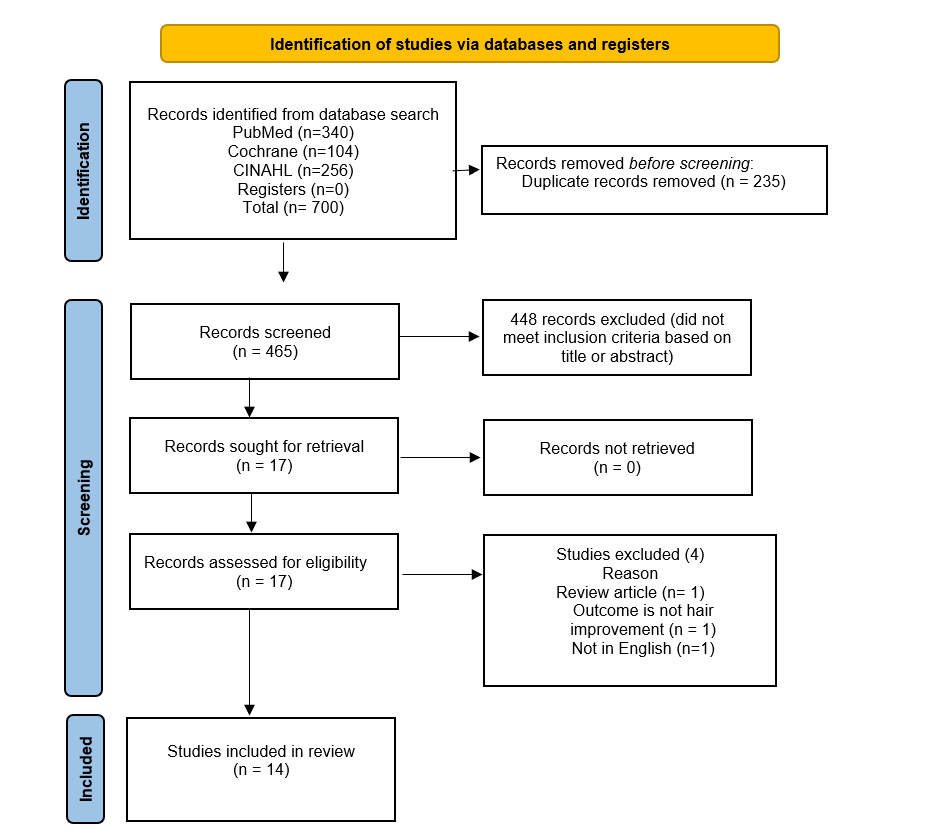

Supplement: Supplementary file 1 — Figure S1: PRISMA flow diagram showing the study selection process. [file JOCD-25-e70817-s001.jpg]
