# Supplementary material for: Evaluating the Effectiveness of Commercial Oral Supplements for Hair Growth: A Systematic Review and Meta‐Analysis
Source: J Cosmet Dermatol. 2026 Apr 9;25(4):e70817. doi: 10.1111/jocd.70817 (PMC13063204; doi:10.1111/jocd.70817)
Supplement: Supplementary file 2 — Table S1: Detailed characteristics of the 14 included studies, including study design, population, intervention, comparators, and study duration. Table S2: Summary of efficacy findings, patient‐reported outcomes (PROs), and safety profiles of the evaluated oral nutraceuticals. [file JOCD-25-e70817-s002.docx]

Supplementary Table 1. Study characteristics of included studies

| Authors | Year | Country | Study Design | Sample Size | Population | Intervention / Exposure | Comparator | Way to Take Pill | Duration |
| --- | --- | --- | --- | --- | --- | --- | --- | --- | --- |
| Dudonné et al. (24) | 2024 | England | RCT | 66 (65) | Women 24–64 y, telogen ≥15% | 15 mg wheat polar lipids (Ceramosides™) | Placebo | 2 capsules every evening before sleeping | 84 days |
| Bhatia et al. (25) | 2025 | England | RCT, double-blind, placebo-controlled | 112 (85) | Men 21–61 y, hair thinning | Nutrafol Men’s Capsules | Placebo | Four capsules once daily with a meal | 180 days (6 mo) |
| Thom (26) | 2006 | England | RCT, double-blind, placebo-controlled | 55 (55) | Adults ≥18 y with hair-loss | 2–3 tabs Nourkrin® daily (weight-based) | Placebo | 2 tabs (< 80 kg) or 3 tabs (> 80 kg) with meal | 6 mo blinded; 12 mo open |
| Milani et al. (27) | 2023 | Italy | Prospective, randomized, assessor-blinded trial | 83 (76) | AGA/FAGA or chronic TE, ≥18 y | GFM oral supplement | Drug alone | 1 tablet daily per oral | 12 weeks |
| Piquero-Casals J et al. (28) | 2025 | Switzerland | RCT, double-blind, placebo-controlled | 80 (80) | AGA/CTE, males & females 18–60 y | Lambdapil®-ISDIN | Placebo | 1 capsule daily orally | 6 months |
| Sivamani et al. (29) | 2024 | USA | Multicenter, single-blind prospective study | 110 (95) | Women 18–60 y, healthy | Nutrafol Women’s Vegan | None | 4 capsules daily | 6 months |
| Nobile et al. (30) | 2025 | Italy | Multicenter, randomized, placebo-controlled | 131 (99) | Women 18–65 y, acute TE | Ceramosides™ oil (35 mg) & powder (15 mg) | Placebo | 2 capsules every evening | 84 days |
| Ablon and Kogan (31) | 2021 | USA | RCT, double-blind, placebo-controlled + open-label | 70 (60) | Women 40–65 y with thinning of hair | Nutrafol Women’s Balance (1 cap/day) | Placebo | 1 capsule daily with a meal | 6 mo + 6 mo open |
| Nichols et al. (32) | 2017 | USA | Open-label, prospective, proof-of-concept study | 10 (10) | Adults aged 18 to 65 years of age with a diagnosis of AGA | Forti5® | None | 2 Tablets daily | 24 weeks |
| Ablon and Kogan (33) | 2018 | USA | Randomized, Double-Blind, Placebo-Controlled Study | 26 active, 14 placebo | Healthy women 21–65 y, Fitzpatrick I–IV, thinning hair | Novel Nutrafol Women’s Capsules | Placebo | 4 capsules once daily per oral | 180 days |
| Ablon (34) | 2016 | England | RCT, double-blind, placebo-controlled | 60 (60) | Men with MPHL | Viviscal® Man (2 tabs/day) | Placebo | 1 tablet AM & PM after meal | 180 days |
| Ablon (35) | 2015 | USA | RCT, double-blind, placebo-controlled | 60 (60) | Women 21–65 y, Fitzpatrick I–IV, thinning | Viviscal Extra Strength | Placebo | 1 tablet × 2 daily after meal | 3 months |
| Beer et al. (36) | 2014 | Italy | RCT, parallel-group, double-blind, placebo-controlled | 50 (50) | Women 40–71 y, stressed/damaged hair | Cynatine HNS (keratin + zinc + B-vitamins) | Placebo (inactive ingredients maltodextrin 370 mg and magnesium stearate 5.0 mg) | Two capsules daily after breakfast | 90 days |
| Lassus and Eskelinen (23) | 1992 | England | RCT, parallel-group | 40 (40) | Men 20–30 y, AGA | Viviscal® vs fish extract | Fish extract | Two tablets twice daily | 6 months |

Supplementary Table 2. Summary of Efficacy, Patient-Reported Outcomes, and Safety of Oral Nutraceuticals for Hair Growth in Included Studies

| Authors | Outcome Measures | Efficacy Findings | PROs & QoL | Safety / Comments | Brand/Product Name |  |
| --- | --- | --- | --- | --- | --- | --- |
| Dudonné et al. (24) | | Phototrichogram; pull test; hair diameter and growth; VAS; sebum | Telogen ↓23.5% vs ↓8.3%; Anagen ↑11.9% vs ↑5.6%; Pull test −47.9% vs −38.2%; Hair growth +12.5% vs none | VAS improved in 64% vs 53% regarding hair density and 64% vs 59% in volume; satisfaction ↑ | None | Ceramosides™ |
| Bhatia et al. (25) | | Physical exam; global photos; pull test; MHGQ; ASEX; PSS; QoL | IGA growth improved 79% vs 51% (p<0.01); IGA quality 74% vs 54% (p=0.026); slowed loss 85% vs 55% | 83% vs 66% showed satisfaction; no ASEX change; PSS: significant reduction in intervention group (p < 0.01 vs. baseline) | NA | Nutrafol Men's Capsules, Nutraceutical Wellness Inc., New York, NY, USA |
| Thom E (26) | | Hair counts; VAS satisfaction | Hair count ↑35.7% vs ↑1.7% (p<0.001) | VAS 5.9 vs 0.6 at 6 month | NA | Nourkrin® (Pharma Medico International, Aarhus, Denmark) |
| Milani et al. (27) | | GAS score; QoL; tolerability | ΔGAS +1.67 vs +0.66 (95% CI 0.52–1.50); GAS≥0 in 85.4% vs 48.6% | “Good/very good” 90.9% vs 78.6% | 86.4% vs 94.1% | GFM oral (Cantabria Labs Difa Cooper, Caronno Pertusella, Italy) |
| Piquero-Casals J et al. (28) | | Hair density & anagen; volume; shedding; QoL | Density +12.3 vs +1.5 hairs/cm²; Anagen +21.4 vs +1.9; Volume +19.2 vs +1.3 cm² (all p<0.001) | No significant difference | No moderate or severe adverse events | Lambdapil 5 alfa plus® |
| Sivamani et al. (29) | | Hair parameters; shedding; strength | Terminal +15%; Total +12.7%; Width +2.3 mm; Shedding −50% | 87–97% self-rated improvement | NA | Nutrafol Women's Vegan Capsules, Nutraceutical Wellness, Inc., New York, NY |
| Nobile et al. (30) | | Phototrichogram; pull test; elongation; growth; self-assessment | Telogen ↓26.9%/24.2% vs ↓11.0%; Anagen ↑10.3%/10.8% vs +5.4%; Growth +16.7%/11.3% vs placebo (P<0.01) | WPLC-O : 93.9%, WPLC-P : 97.0% reported lost less hair | None | Ceramosides™ (oil & powder) France |
| Ablon and Kogan (31) | | Hairs (terminal/vellus); shedding; global assessments; QoL | Terminal +10.2% vs +0.7% (p<0.01); Total +9.97% vs –0.2%; vellus hair 10.78% vs -3.38%; Shedding −32.4% vs −10.1% | 30% reported moderate improvement in overall hair growth, hair volume and scalp coverage at day 180 , while its 0% in placebo group | NA | NUTRAFOL® Women's Balance, Nutraceutical Wellness Inc., NewYork, NY |
| Nichols et al. (32) | | Hair Mass Index (HMI); Terminal and vellus hairs; Investigator Global Photography Assessment | 80% of participants showed improvement; HMI 9.5% increase;  Terminal 5.9% increase | Global photography assessment:  40% were moderately increased | No AEs | Forti5® (Q-SkinScience®, Miami, Florida) |
| Ablon and Kogan (33) | | Terminal/vellus/total hair counts; global assessments; diameter; SAQ; ease-of-use; QOL; AEs | At D180 vs placebo: terminal +10.4% vs +3.5%; vellus +15.7% vs –2.2%; total +10.8% vs +3%; growth score +1.08 vs +0.08 (p = 0.016) | Quality +1.12 vs +0.08 (p = 0.005); hair diameter no change; SAQ: 73–81% report growth/volume/thickness improvement; ease-of-use 84.6%; | No treatment-related AEs | Nutrafol® Women's Capsules |
| Ablon (34) | | Photography; pull test; QoL; self-assess | Count ↑162.2→174.9; Density ↑159.7→172.2; Pull test ↑ (p<0.01) | QoL improved on 3/6 questions | N/A | Viviscal® Man, Lifes2good, Inc., Chicago, IL, USA |
| Ablon (35) | | Phototrichograms; shed hairs; SAQ; QoL; safety | Terminal ↑178.3→235.8 vs 180.9; Vellus ↑; Shedding ↓ | Higher SAQ & QOL vs placebo | No AEs | Viviscal® Extra Strength |
| Beer et al. (36) | | Pull test; anagen/telogen; tensile strength; appearance | Pull test +46.6%; Anagen +9.2%; Tensile +5.9%; Appearance +1.10 | NA | No AEs | Cynatine HNS |
| Lassus and Eskelinen (23) | | Punch biopsy; non-vellus hair counts | Non-vellus hairs +38.1% vs +2.1%; 19/20 no longer histologically alopecic | NA | No AEs | Viviscal® |
